# Puncture accuracy of an optical tracked robotic aiming device—a phantom study

**DOI:** 10.1007/s00330-022-08915-z

**Published:** 2022-06-09

**Authors:** Yannick Scharll, Sofia Letrari, Gregor Laimer, Peter Schullian, Reto Bale

**Affiliations:** Interventional Oncology-Microinvasive Therapy (SIP), Department of Radiology, Medical University Innsbruck, Anichstr. 35, 6020 Innsbruck, Austria

**Keywords:** Robotics, Oncology, Imaging, Tomography, X-ray computed, Radiology, Interventional

## Abstract

**Objectives:**

To evaluate the targeting accuracy of stereotactic punctures based on a hybrid robotic device in combination with optical tracking—a phantom study.

**Methods:**

CT data sets of a gelatin-filled plexiglass phantom with 1-, 3-, and 5-mm slice thickness were acquired. An optical navigation device served for planning of a total of 150 needle trajectories. All punctures were carried out semi-automatically with help of the trackable iSYS-1 robotic device. Conically shaped targets inside the phantom were punctured using Kirschner wires. Up to 8 K-wires were positioned sequentially based on the same planning CT and placement accuracy was assessed by taking control CTs and measuring the Euclidean (ED) and normal distances (NDs) between the wire and the entry and target point.

**Results:**

Using the StealthStation S7, the accomplished mean ND at the target for the 1-mm, 3-mm, and 5-mm slice thickness was 0.89 mm (SD ± 0.42), 0.93 mm (SD ± 0.45), and 0.73 mm (SD ± 0.50), respectively. The corresponding mean ED was 1.61 mm (SD ± 0.36), 2.04 mm (SD ± 0.59), and 1.76 mm (SD ± 0.45). The mean duration of the total procedure was 27.9 min, including image acquisition, trajectory planning, registration, placement of 8 wires, and the control-CT.

**Conclusions:**

The optically tracked iSYS-1 robot allows for precise punctures in a phantom. The StealthStation S7 provided acceptable results and may be helpful for interventions in difficult anatomical regions and for those requiring complex multi-angle trajectories. In combination with our optical navigation tool, the trackable robot unit allows to cover a large treatment field and the compact design facilitates placement of needle-like instruments.

**Key Points:**

*• The use of a robotic targeting device in combination with optical tracking (hybrid system) allows for accurate placement of needle-like instruments without repeated control imaging.*

*• The compact robotic positioning unit in combination with a camera for optical tracking facilitates sequential placement of multiple K-wires in a large treatment volume.*

## Introduction

Stereotaxy plays an important role in interventional radiology. High precision is essential for the success of stereotactic procedures, widely used for diagnostic and therapeutic purposes. The standard procedure includes computerized 3D planning of needle paths at arbitrary angulations and orientations after preinterventional 3D imaging [1–3]. New tools for percutaneous procedures face the challenge to make punctures of almost every anatomical structure feasible. In conventional navigation systems, the interventionalist introduces the probe after manual alignment of the aiming device [4]. On the other hand, alignment with the virtual needle path can also be carried out (semi-) automatically by robot-assisted systems. Stoffner et al [5] performed several punctures in a CT environment to compare the performance of a robotic system to a manual aiming device. The handling of both systems is different. The robotic assistance system aligns automatically with the dedicated needle path, whereas the Atlas has to be positioned manually under real-time guidance using an optical navigation system. Although both systems yielded comparable accuracy, additional inaccuracies can occur during manual trajectory alignment (e.g., jitter). Robotic devices are less operator-dependent and can overcome human physiological fatigue but face other limitations. In fact, some systems are large in size and have a lacking flexibility in selecting different entry points [6]. Positioning of multiple probes is often limited due to a restricted working range of the end-effector. When several targets have to be punctured, e.g., for radiofrequency ablation of large or multiple liver tumors, it is challenging to avoid collision with other needles in place. Kettenbach et al [7] used a robotic needle-guidance platform, the iSYS-1, to assess the feasibility during CT-guided punctures in a phantom. In comparison to other commercially available systems, the design of the robotic positioning unit (RPU) is compact. Radiopaque markers mounted to the RPU of the iSYS-1 have to be included in the planning CT to allow for automatic registration. If the pre-positioning of the RPU deviates too much from the target, readjustment might be necessary, requiring an additional CT scan to register the new position of the tool and the target. In fact, the range of translation and angulation of the needle guide is limited.

To overcome this limitation, optical and/or electromagnetic systems can be used for continuous localization of medical tools with respect to the patient anatomy [8]. In fact, optical tracking of the RPU would allow to reposition the robotic unit under continuous deviation-to-trajectory feedback without need of a new CT scan. The increase in flexibility of this hybrid approach could be promising for interventions covering a large treatment volume and for those requiring multiple probes placements. Hence, the aim of this study was to test the accuracy of the iSYS-1 in combination with an optical navigation tool by in vitro phantom tests.

## Material and methods

### Phantom

**Fig. 1:**
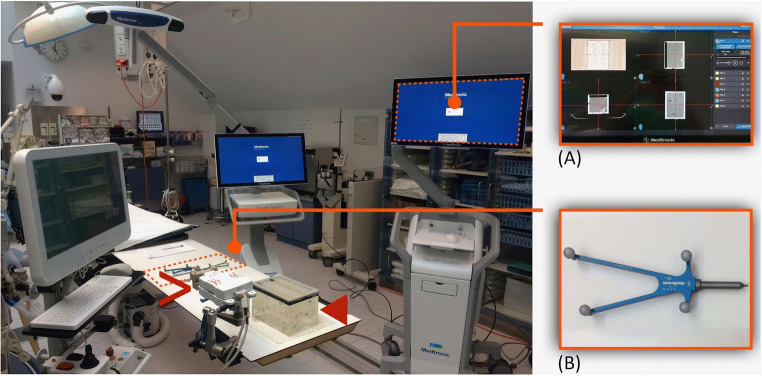
Experimental setup showing the navigation system with its planning monitor (**A**), the iSYS-1 robot unit (>) and the plexiglass phantom (◄) filled with gelatin. The Vertek probe (**B**), a passive guidance tool with 4 spherical reflective markers for optical tracking and precise trajectory alignment

The phantom (Fig. 1) used for this study consists of a plexiglass box (220 × 150 × 175 mm) with a removable cover with 28 holes drilled in it, measuring 8 mm in diameter. Inside the cube we find 8 conically shaped target markers, having aluminum tips with a normal distance of up to 130 mm from the lid. By flipping and rotating the lid, 112 individual entrance points can be chosen. The phantom is filled with gelatin in order to withhold the K-wires for CT evaluation. The same phantom was previously used for accuracy analysis of other guidance systems [5, 9, 10].

### iSYS-1 robotic-assisted needle placement

**Fig. 2:**
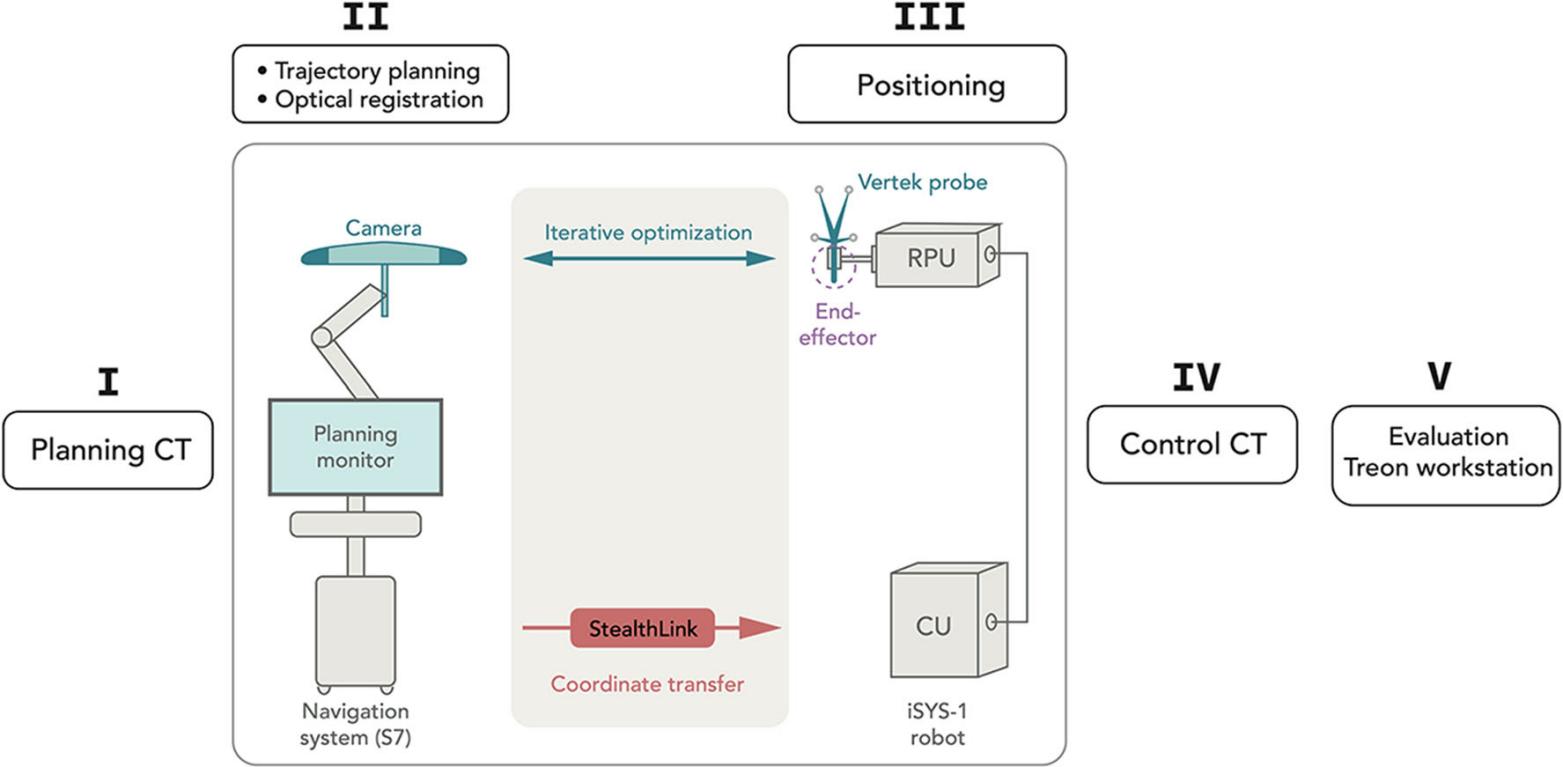
Operational flow of the hybrid system. The navigation system communicates with the robot control unit (CU) via interface (StealthLink). I: Transfer of DICOM formatted images from the CT console to the navigation system. II: Sequential planning of up to 8 trajectories during the same work step, followed by marker-based optical registration of the phantom. III: Positioning of the guidance sheath (further explained in Fig. 3) and insertion of up to 8 K-wires sequentially. Detection of the Vertek probe via camera allows for iterative optimization during trajectory alignment. The coordinates are calculated by the navigation system and send via StealthLink interface to the robot. IV+V: The post-procedural control CT scan is transferred to the Treon workstation via the hospitals own intranet for accuracy evaluation

**Fig. 3:**
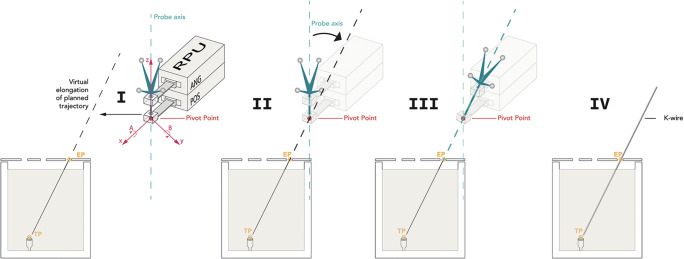
Schematic illustration of the robot positioning process. Cross-sectional view through the phantom showing the planned needle trajectory from the center of the hole inside the cover (entry point, EP) to the aluminum tip (target point, TP). I: At the beginning of the procedure the robot is manually pre-positioned close above the entry point. The software displays, if the current position of the instrument guide allows automatic alignment of the probe axis to the planned trajectory. II: Under continuous deviation-to-trajectory feedback (iterative optimization) of the Vertek probe fine adjustment is automatically executed by the robot. First, the pivot point of the guidance sheath is superimposed with the virtual elongation of the planned trajectory (*X* and *Y* axess). III: Angulation in A and B allows to align for double oblique needle trajectories. IV: The K-wire can be inserted after removing the Vertek probe

The experimental setup is shown in Fig. 2. All needle placements were carried out with the iSYS-1 robotic guidance system (iSYS Medizintechnik GmbH). It consists of a four degrees of freedom robot needle positioning unit mounted to a 7 degrees of freedom passive holding arm, described previously [7]. The four degree of freedom RPU consists of two modules (Fig. 3): a lower part (POS) and an upper part (ANG). POS allows positioning of the end effector in longitudinal axis *X* and in transversal axis *Y*, restricted to a field of 40 × 40 mm (*X* × *Y*). ANG ensures angulation in *A* (rotation around *X* axis) and *B* (rotation around *Y*). Movement of the end effector in the *Z* axis is only possible by hand.

After performing a CT scan of the phantom, datasets were transferred to the navigation system allowing to plan the needle path by selecting the entrance and target point. In this study, the target point was set as the aluminum tip inside the phantom.

### Navigation systems and experimental setup

The planning CT datasets were acquired with 1-, 3-, and 5-mm slice thickness in a Siemens Somatom Sensation Open CT system (Siemens Healthineers) and transferred to the dedicated workstation (Fig. 1(A)).

The needle paths were planned with the navigation device, the StealthStation S7 (S7) (Medtronic Inc.). The system is equipped with a camera for optical tracking of active LED markers on a dynamic reference frame and reflective spherical markers on a navigation probe (Fig. 1(B)). They allow to constantly recognize the orientation of instruments in relationship to the patient’s anatomy or, as in this study, to the phantom. The registration is the most important step in stereotactic and robotic interventions and is based on lead markers (Beekley-Spots) directly attached to the phantom. Marker-based registration with the S7 was accomplished using the Touch-n-go probe (Medtronic). The reference points on the phantom were not automatically recognized by the S7 and had to be defined manually on the planning CT.

**Fig. 4:**
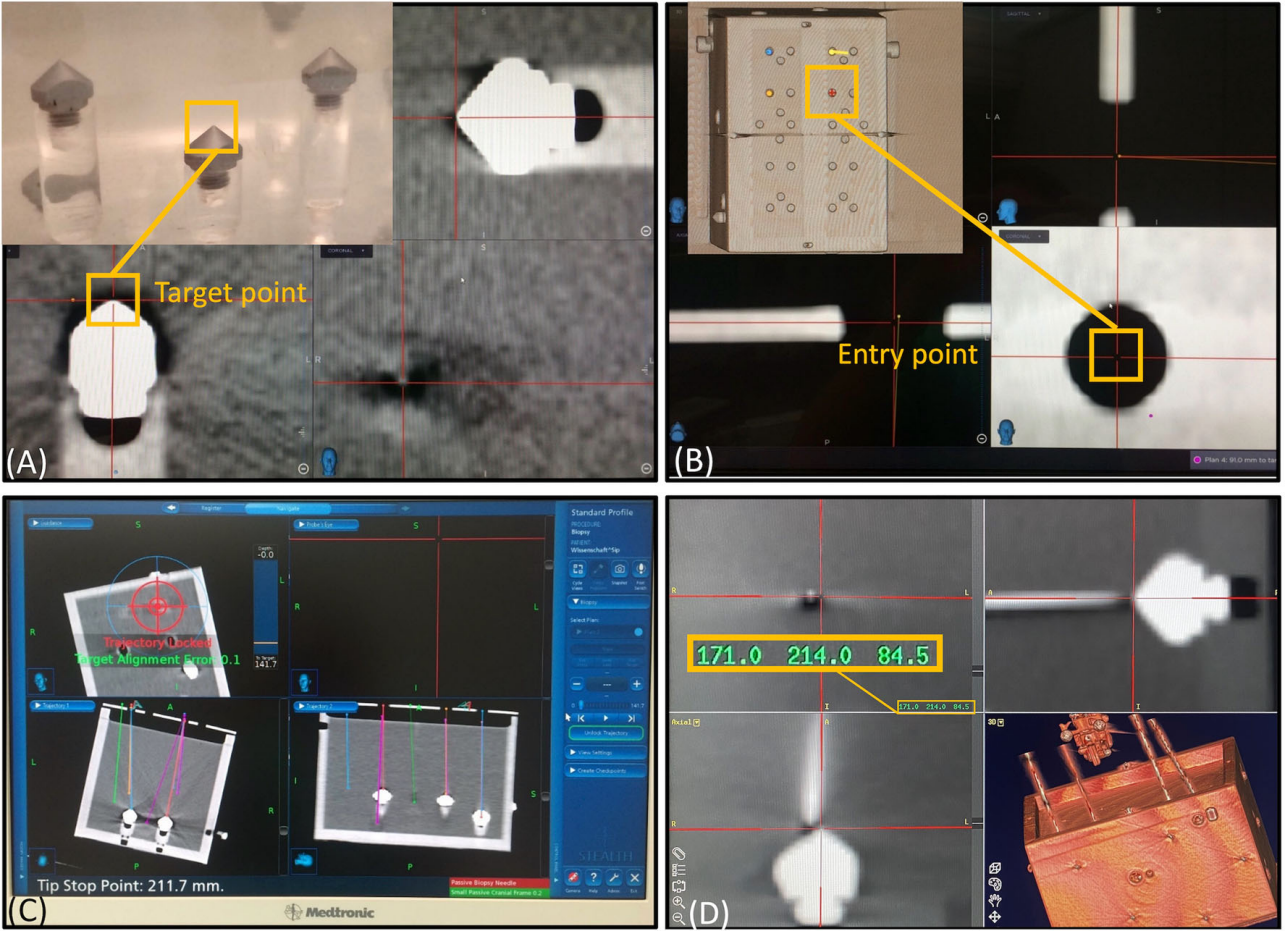
Workstation of the StealthStation S7 (**A**–**C**) during the planning and navigation procedure showing the selected target point (**A**) and entry point (**B**). After clamping the Vertek probe into the needle guide adapter, the robotic unit is pre-positioned over the targeted trajectory by hand. **C** The robotic unit is displayed with circles. Fine adjustment is completed when the circles show concentric. **D** Screenshot of the Treon workstation to evaluate the needle positioning accuracy with the control CT-data. The *x*, *y*, and *z* coordinates (highlighted in the box) were measured by positioning the crosshairs in all three planes. The data was then transferred to an Excel spreadsheet for calculation of the ED and ND

The system requires definition of two fixed points by the operator. Based on multiplanar reformatted images, the target and entry point were selected (Fig. 4a, b) using the software of the optical navigation system. Up to 8 needle paths were planned simultaneously and carried out with robotic guidance.

Upon completion of the trajectory planning and after registration, the S7 software sends the coordinates of the planned trajectory and the actual spatial data of the navigation probe to the iSYS-1 robotic guidance device in real time. The two systems are connected via a specially developed application programming interface (API), named “StealthLink” (Medtronic). The API is a simple query/retrieve interface with static data structures and consists of a C library available for Windows and Linux.

The pre-positioning of the robotic unit near the target was performed under continuous deviation-to-trajectory feedback of the probe (Fig. 4c). After successful registration, positional information of the selected trajectory can be transformed into the robot coordinates and sent to the robot controller. In a final step, the robot unit can be activated and moves automatically to the correct location. The information about the insertion depth is derived from the planning software of the navigation system. The needle is manually introduced according to the preplanned depth.

### Evaluation

**Fig. 5:**
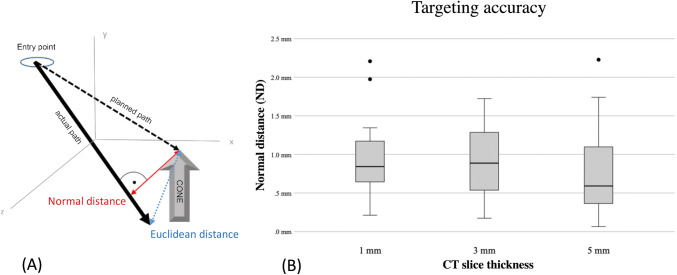
**A** The normal distance describes the shortest possible distance between a point and a straight line. The ED determines the gap between two points in a multidimensional space. It is calculated using the coordinates of the actual position of the needle tip and the target point (dashed arrow), indicating the deviation in the direction of the needle placement. **B** Box plot of the StealthStation S7 displaying the targeting accuracy on the basis of the ND at all slice thicknesses

The post-procedural control CT scans were transferred to the Treon workstation (Medtronic Inc.) and assessed with its “Mach Cranial” software (Fig. 4d). For every probe, the coordinates of the entry point and planned entry point, as well as the tip of the needle and tip of the cone, were determined. The deviations were measured by the calculation of the normal distance (ND) between the target and the wire axis and by the Euclidean distance (ED) between the target and the positioned wire tip (Fig. 5a). The formulas are basic in analytical geometry and can be found in [11].

All statistical analyses were performed using SPSS Version 22 (SPSS Inc.). The distribution of error measurements was graphically checked with histograms and assessed with the Kolmogorov-Smirnov Test. Mean errors, standard deviation, and maximal values as well as minimal values were calculated. Differences between the slice thicknesses were assessed using the independent *t*-test. A *p* value of < 0.05 was considered statistically significant.

Repeatability is related to the standard deviation, and some statisticians consider the two equivalent. The repeatability coefficient (*r*) is the maximum difference that is likely to occur between repeated measurements that are approximately normally distributed and can be defined as$1.96\ x\sqrt{2}\times \mathrm{SD}$ [12].

## Results

In total, 150 punctures were assessed with the S7.

### Accuracy

The mean ED at the target for the 1-mm, 3-mm, and 5-mm slices were 1.61 ± 0.36, 2.04 ± 0.59, and 1.76 ± 0.45 while the corresponding ND was 0.89 ± 0.42, 0.93 ± 0.45, and 0.73 ± 0.50, respectively (Fig. 5b). The repeatability coefficient (*r*) ranges from 1.16 to 1.39 for the ND at the target.

The mean entrance point accuracy (ND) was 0.26 ± 0.20, 0.29 ± 0.28, and 0.42 ± 0.35.

Comparing the accuracy data of the ND at the target by using Student’s *t*-test, only a significant difference was observed comparing the accuracy between the slice thicknesses 3 mm vs. 5 mm.

ND: 1 mm vs. 3 mm, *p* = 0.64; 1 mm vs. 5 mm, *p* = 0.09; 3 mm vs. 5 mm, *p* = 0.037.

### Procedural time

**Fig. 6:**
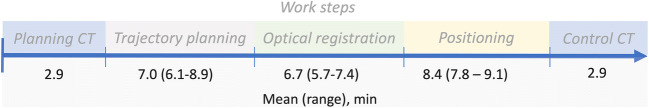
Timeline illustrating the procedural events. The given procedural durations refer to a set of 8 K-wires, being planned and positioned sequentially after one planning CT

The mean duration of the total procedure including image acquisition, trajectory planning, registration, placement of 8 wires, and the control-CT was 27.9 min. A detailed description is given in Fig. 6.

## Discussion

Accuracy and safety are crucial issues of image-guided percutaneous treatments. However, the accuracy of probe placement is usually highly dependent on the physician’s experience.

Stereotactic punctures promise a significant increase in the accuracy of CT-guided needle or probe placement, especially compared with freehand technique. Improved needle placement accuracy usually translates into decreased complication rates, greater sampling success for biopsies, and good oncologic outcomes in terms of local recurrence [13, 14].

### Accuracy

Accuracy is determined by the target registration error (TRE) and the target positioning error (TPE) [15]. For the S7, the manual registration of the markers provided a TRE of 0.3 mm for a CT slice thickness of 1 mm. This error contains technical errors in the computer-assisted position measurement, errors in definition of markers, image errors in indicating the real markers, and general software registration errors. The TPE is specified by the ED (TPE-total) and ND (TPE-lateral).

All 150 punctures were performed without technical failure. The iSYS 1-robot in combination with the S7 yielded reproducibly and accurate results in a millimeter/submillimeter range and the repeatability coefficient is low. In theory, the aluminum tip may avoid overshooting the target. However, the conical shape of the target body rather leads to an additional lateral deviation due to deflection at the tip. Therefore, it may rather lead to an artificial increase of the normal distance than an underestimation of the Euclidean distance (ED). In clinical practice, overshooting the target can either be prevented by performing a control-CT close to the target or easily be corrected by retracting the needle. An angular deviation of the wire is more critical since it may require manual reangulation or complete reinsertion. For this reason, the normal distance (ND) seems to be the most important factor.

At the expense of a higher radiation dose, the best results for the ED are achieved with the 1-mm slice thickness. On the other hand, the best results for ND at the target are achieved with the 5-mm slice thickness. In fact, the slightly better results at 5 mm are mainly explainable due to general and technical errors (e.g., different gel consistency), yet there is no significant difference comparing them with the 1-mm layer (*p* = 0.09). The aiming device did not show a significant difference when comparing the ND targeting accuracy at 1 mm vs. 3 mm. Therefore, an almost similar puncture accuracy with lower radiation dose can be achieved.

### Comparison with previous studies and other guidance devices

A comparable phantom study was performed by Kettenbach et al [7], revealing accurate needle placement using the iSYS-1 equipment. The ED between the actual needle tip and the target was 2.3 ± 0.8 mm. The study was limited to a CT slice thickness of 1 mm and 10 needle placements, planned on a laptop. In contrast to our setup, readjustment of the robotic unit was not possible once the planning CT was made. The same limitation occurred to the study investigated by Groetz et al [16] where the iSYS-1 was used in combination with the RoboNav-Software by MedCom. Markers directly attached to the needle guide had to be visible in the planning CT not allowing to move the robotic unit subsequently. For both studies, it was important to mount the iSYS-1 with approximate prior knowledge of the target. This is critical, because in a clinical routine inaccurate pre-positioning needs readjustment of the needle holder, necessarily followed by a new CT scan to register the new position of the tool and the target.

In our study, application of the iSYS-1 in combination with the optical system was feasible in all cases with a short learning curve. Even trajectories at extreme angles and deep locations were possible. Double oblique punctures with a target depth up to 130 mm could be accomplished even though the robot is restricted to a field of 40 × 40 mm. If a higher number of probes is required, readjustment of the robotic unit is necessary. The use of optical tracking allowed repositioning of the robotic unit under continuous deviation-to-trajectory feedback without need of new CT scans.

Our group tested different navigation systems with the identical phantom. As an example, Stoffner et al [5] tested the Innomotion, a robotic assistance system, showing accurate results comparable to the iSYS-1. Moreover, the same phantom was used by Putzer et al [9] and Venturi et al [17], investigating the accuracy of two electromagnetic navigation systems, the AxiEM and PercuNav, as well as the accuracy of the low-cost targeting system ArciNav (Table 1).

**Table 1:** Comparison of the accuracy at the target of the iSYS-1 to the previously reported results of our group. Venturi et al [19] investigated the accuracy of the ArciNav, Stoffner et al [5] used the Stealth Station Treon and the Innomotion, and Putzer et al [9] tested the AxiEM and the PercuNav. All postprocedural control CT scans were performed in 1 mm slice thickness

	iSYS-1 S7	ArciNav	Stealth Station Treon	Innomotion	AxiEM	PercuNav
**1mm**						
ED mean (mm)	1.606	2.52	1.94	1.69	3.855	4.417
ED min. (mm)	0.757	0.66	0	0.53	1.075	2.441
ED max. (mm)	2.476	4.6	4.79	3.25	15.775	8.699
ED standard dev. (mm)	0.363	0.64	0.912	0.772	2.275	1.333
ND mean (mm)	0.885	1.42	1.64	1.42	3.291	3.759
ND min. (mm)	0.213	1.22	0	0.18	0.259	1.272
ND max. (mm)	2.207	2.55	4.57	3.07	9.871	8.699
ND standard dev. (mm)	0.422	0.66	0.919	0.78	1.517	1.594
**3mm**						
ED mean (mm)	2.042	2.8	2.2	1.91	3.744	4.263
ED min. (mm)	0.728	0.53	0.63	0.95	1.050	2.062
ED max. (mm)	3.019	5.26	5.54	3.87	10.327	7.974
ED standard dev. (mm)	0.452	1	1.136	0.673	2.101	1.322
ND mean (mm)	0.927	1.99	1.84	1.60	3.157	3.840
ND min. (mm)	0.174	0.18	0.09	0.16	0.441	1.029
ND max. (mm)	1.722	5.24	5.09	3.73	8.428	7.723
ND standard dev. (mm)	0.450	1.1	1.189	0.733	1.522	1.425
**5mm**						
ED mean (mm)	1.763		2.74	2.30	4.813	4.457
ED min. (mm)	0.601		0.31	0.66	1.342	2.095
ED max. (mm)	2.875		6.84	5.17	10.467	9.217
ED standard dev. (mm)	0.452		1.166	0.881	2.065	1.562
ND mean (mm)	0.726		2.48	1.98	3.934	3.807
ND min. (mm)	0.065		0.12	0.52	0.110	0.496
ND max. (mm)	2.227		6.64	5.17	8.757	8.103
ND standard dev. (mm)	0.497		1.196	1.002	1.683	1.708

Overall, the iSYS-1 in combination with the optical navigation system turned out to be the most accurate system tested by our group.

Other robotic devices have been presented in the past. Croissant et al [18] evaluated the accuracy of a robotic interventional assistance platform (Perfint Healthcare: Maxio) in a cadaver study, revealing highly accurate results using during spinal interventions. The robot-assisted placement of 24 K-wires showed a mean deviation of 1.2 mm in the horizontal axis and a mean deviation of 0.5 mm in the vertical axis. The system has to be mounted on a special registration plate at the CT table side and is quite large with an unwieldy end effector, making it difficult to place multiple probes on a small treatment field. In contrast, the compact design of the iSYS-1 facilitates selecting different entry points.

### Limitations

The results of the phantom study can only be partially applied to clinical practice. The phantom filling is a homogeneous mass not reflecting the human body with its different tissue consistencies. In the clinical setting, patient movements, different kinds of tissue properties, anatomical position of lesions, and respiratory motion have to be taken into account and may result in larger errors. Holzknecht et al [19] tested a virtual CT puncture system with skin sensors and came to the result that the patient’s breathing movement is an important limiting factor resulting in deviations of up to 20 mm. To overcome this limitation, they performed 50 diagnostic punctures using an electromagnetic guidance system and the respiratory movements of the patient were recorded and displayed. The biopsy needle was registered with two sensors by electromagnetic detection, allowing for virtual real-time navigation with high accuracy. Widmann et al [20] reported on temporary endotracheal tube disconnections as a safe and effective technique for respiratory motion control during image-guided interventions. Others have suggested anesthetic maneuvers with use of jet ventilation, proving to be a feasible, safe, and radiation dose–reducing method [21].

In conclusion, the iSYS-1 in combination with an optical tracking system offers accurate needle placement. Our hybrid setup provides accurate needle guidance, even for double oblique angulated approaches. The additional use of optical tracking facilitates multi-probe needle placements in a large treatment volume. Therefore, we believe that this device will be helpful for interventions with difficult anatomical conditions and complex multi-angle trajectories. However, further in vivo studies will be required in order to define its role in clinical practice.

## Abbreviations

CU: Control unit
ED: Euclidean distance
K-wires: Kirschner wires
ND: Normal distance
RPU: Robotic positioning unit
S7: StealthStation S7
TPE: Target positioning error
TRE: Target registration error

## Declarations

### Guarantor

The scientific guarantor of this publication is Dr. Reto Bale.

### Statistics and biometry

No complex statistical methods were necessary for this paper.

### Methodology

• Performed at one institution
